# Systemic arteriosclerosis is associated with left ventricular remodeling but not atherosclerosis: a TASCFORCE study

**DOI:** 10.1186/s12968-018-0428-0

**Published:** 2018-01-30

**Authors:** Jonathan R. Weir-McCall, Matthew Lambert, Stephen J. Gandy, Jill J. F. Belch, Ian Cavin, Shelley A. Henderson, Roberta Littleford, Jennifer A. Macfarlane, Shona Z. Matthew, R. Stephen Nicholas, Allan D. Struthers, Frank Sullivan, Richard D. White, J. Graeme Houston

**Affiliations:** 1 Division of Molecular and Clinical Medicine, College of Medicine, University of Dundee, Level 7, Dundee, DD1 9SY UK; 2 NHS Tayside Medical Physics, Ninewells Hospital, Dundee, UK; 3 Department of Research and Innovation, North York General Hospital, University of Toronto, Toronto, Canada; 4 Department of Clinical Radiology, University Hospital of Wales, Cardiff, CF14 4XW UK

**Keywords:** Arterial complaince, Arteriosclerosis, Atherosclerosis, Left ventricle, Cardiovascular risk

## Abstract

**Background:**

Arteriosclerosis (arterial stiffening) is associated with future cardiovascular events, with this effect postulated to be due to its effect on cardiac afterload, atherosclerosis (plaque formation) progression or both, but with limited evidence examining these early in disease formation. The aim of the current study is to examine the association between arteriosclerosis, atherosclerosis and ventricular remodelling in a population at low-intermediate cardiovascular risk.

**Methods:**

One thousand six hundred fifty-one subjects free of clinical cardiovascular disease and with a < 20% 10 year cardiovascular risk score underwent a cardiovascular magnetic resonance (CMR) study and whole body CMR angiogram. Arteriosclerosis was measured using total arterial compliance (TAC) - calculated as the indexed stroke volume divided by the pulse pressure. Atherosclerosis was quantified using a standardised atheroma score (SAS) which was calculated by scoring 30 arterial segments within the body based on the degree of stenosis, summating these scores and normalising it to the number of assessable segments. Left ventricular remodelling was measured using left ventricular mass to volume ratio (LVMVR).

**Results:**

One thousand five hundred fifteen (38% male, 53.8 ± 8.2 years old) completed the study. On univariate analysis TAC was associated with SAS but this was lost after accounting for cardiovascular risk factors in both males (B = − 0.001 (− 0.004–0.002),*p* = 0.62) and females (B = 0.000(95%CI -0.002--0.002),*p* = 0.78). In contrast compliance correlated with LVMVR after accounting for cardiovascular risk factors (B = − 0.12(95%CI -0.16--0.091),*p* < 0.001 in males; B = − 0.12(95%CI -0.15--0.086),*p* < 0.001 in females).

**Conclusion:**

Systemic arteriosclerosis is associated with left ventricular remodelling but not atherosclerosis. Future efforts in cardiovascular risk prevention should thus seek to address both arteriosclerosis and atherosclerosis individually.

**Electronic supplementary material:**

The online version of this article (10.1186/s12968-018-0428-0) contains supplementary material, which is available to authorized users.

## Background

Arteriosclerosis is the stiffening of the arterial wall, which occurs with advancing age and is strongly associated with major adverse cardiovascular events [1]. It is predominantly a product of age and pulse pressure, reflecting the repetitive strain of the pulsatile cardiac output on the elastic fibres of the arterial wall. While its association with cardiovascular morbidity and mortality is well established, the exact pathophysiological mechanisms by which it exerts this effect are not entirely clear [2]. The key pathophysiological process underpinning cardiovascular disease is the formation, progression and rupture of atherosclerotic plaque. Increased arterial stiffness has been linked to reduced wall shear stress and thereby may be an antecedent event to the formation of atherosclerotic plaques [3]. Alternatively it may be that, other than the aging process, arteriosclerosis and atherosclerosis are autonomous risk variables, exerting independent adverse effects on the cardiovascular system and thus requiring individual therapeutic targetting.

Previous work has demonstrated mixed results between coronary artery calcification and carotid plaque and arterial stiffness [4–6]. However a limitation of these techniques is their focus on a single arterial territory. Atherosclerosis is a systemic disease, thus absence of plaque in one territory does not equate to absence of plaque in another [7, 8]. Whole body cardiovascular magnetic resonance angiography (WB-CMRA) allows systemic quantification of the global atherosclerosis burden within the body, with the results of this showing strong prognostic capabilities for future adverse cardiovascular events [9–11]. Thus the aim of the current study was to compare early atherosclerotic plaque formation and arterial stiffening in a non-high risk cohort with no prior diagnosis of cardiovascular disease, to test the hypothesis that increased arterial stiffening would be associated with an increased atherosclerotic plaque burden.

## Methods

### Study population

**Fig. 1:**
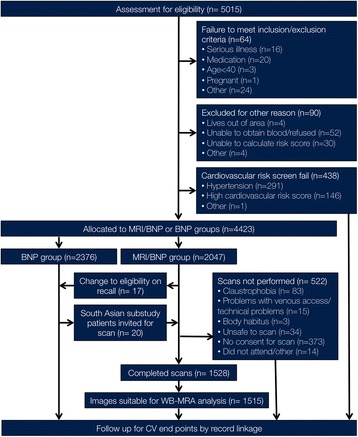
CONSORT diagram for the TASCFORCE study

Four thousand four hundred twenty-three participants were recruited to the Tayside Screening for Cardiovascular Events (TASCFORCE) study from which 2047 subjects were recruited to the imaging arm (see CONSORT diagram (Fig. 1) for full details) [12]. Subjects were enrolled if they were: (i) ≥40 years old; (ii) clinically and symptomatically free of cardiovascular disease; (iii) had a serum brain natriuretic peptide (BNP) level greater than their gender specific median; and (iv) had a ten-year risk of coronary heart disease < 20% as predicted by the Adult Treatment Panel III (ATPIII) algorithm [13]. Exclusion criteria included: (i) pregnancy; (ii) known cardiovascular disease; (iii) known primary muscle disease; (iv) active liver disease (these latter two due to a proposed later planned study with statins); (v) known diabetes; (vi) other known illness or contraindication to CMR; (vii) participation in a clinical trial; (viii) inability to give informed consent; (ix) known alcohol abuse; and (x) a blood pressure of greater than 145/95 mmHg.

### Image acquisition

The CMR protocol has been described in detail elsewhere [14], but in brief, imaging was performed using a 3 T system (Magnetom Trio, Siemens Healthineers, Erlangen, Germany). WB-CMRA was performed using a dual bolus injection technique a CMR cine sequences performed before the first contrast injection.

**Fig. 2:**
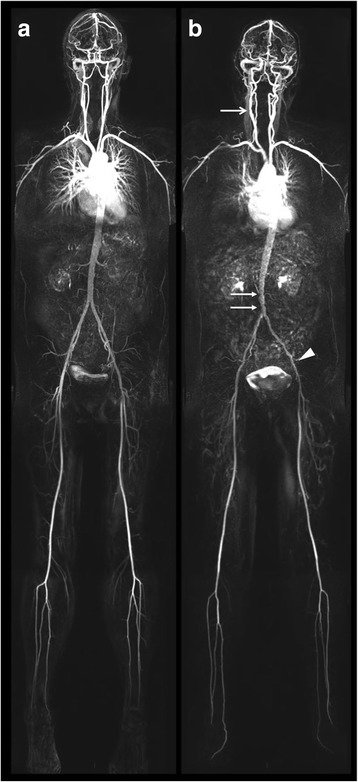
Whole body cardiovascular magnetic resonance angiograms (WB-CMRA) demonstrating  (**a**) a normal study free from atherosclerosis (**a**) and **b**) a study with extensive low grade atherosclerosis with stenosis of the right carotid bulb (open arrow head), aorta (closed arrow head) and left external iliac artery (triangle)

For WB-CMRA acquisition, the body was divided into 4 stations to cover the entire arterial tree: 1) Head, neck and thorax; 2) Abdomen and pelvis; 3) Thighs; and 4) Peripheral run off. Following the injection of the first bolus of 10 ml of 0.5 mmol/ml gadoterate meglumine (Dotarem, Guerbet, Villepinte, France) followed by 20 ml saline injected at a rate of 1.5 ml/s, acquisition of the head, neck and thoracic station was acquired using a 3D TurboFLASH sequence. This was rapidly followed by three sequential acquisitions of the calf vessels after completion of the thoracic station to account for variable arterial transit times to the distal leg vessels. Following a short delay the second dose of 15 ml of gadoterate meglumine was infused at 1.5 ml/s followed by a 20 ml saline flush with acquisition of the two stations covering the abdominopelvic and thigh vessels. Figure 2 demonstrates examples of whole body angiograms of two participants with a normal standardized atheroma score (SAS) and a high SAS.

For CMR, a body matrix radiofrequency (RF) coil (6 elements) was used in combination with a spine array (up to 24 elements). ECG-gated segmented breath-hold cinematic (cine) TrueFISP images were acquired in the horizontal and vertical long axes, and in the short axis from the atrio-ventricular ring to the LV apex using 2D ECG-gated breath-hold segmented cine TrueFISP sequence.

### Image analysis

WB-CMRA images were analyzed by one of four blinded observers on standard image analysis workstations (using Carestream PACS v10.1 on Barco 3MP monitors, Barco, Belgium) using the original source post-contrast images, along with subtracted multiplanar reformats and maximum intensity projections. The whole-body arterial tree for every patient was divided anatomically into 31 segments: right and left internal carotid arteries; right and left vertebral arteries; right and left common carotid arteries; innominate artery; right and left subclavian arteries; aortic arch; thoracic aorta; abdominal aorta; coeliac artery; superior mesenteric artery; inferior mesenteric artery; right and left renal arteries; right and left iliac arteries; right and left femoral arteries; right and left profunda femoris arteries; right and left popliteal arteries; right and left anterior tibial arteries; right and left peroneal arteries; and right and left posterior tibial arteries. Each arterial segment was visually assessed for the region of greatest stenosis. According to the degree of this stenosis each vessel was scored from 0 to 4: 0 = Disease-free vessel; 1 = < 50% stenosis; 2 = 50–70% stenosis; 3= > 70% stenosis; and 4 = Vessel occlusion. Arterial segments which were not visualised with sufficient clarity for grading of the degree of stenosis were not analysed.

Atherosclerotic plaque burden was quantified using a standardised atheroma score (SAS). To calculate this, the score of the 30 arterial segments was summated and then, to account for the fact that not all vessels could be scored in all participants, the final score was divided by the number of segments which had been successfully analyzed (n), and then calculated as a percentage of the maximum possible stenosis score (see equation below) [15].

$$ SAS=\left[\left(\frac{\Sigma \kern0.5em vessel scores}{n}\kern0.5em \right)\times \frac{1}{4}\right]\times 100 $$

The reproducibility of this scoring technique has been previously reported based on the analysis of 48 scans randomly selected by the trial statistician with excellent intra- and inter-observer agreement [14].

Left ventricular (LV) mass and volume quantification was performed as previously described [14]. LV mass and volumes were indexed to body surface area (calculated using the Dubois and Dubois formula). LV mass: volume ratio (LVMVR) was calculated using non-indexed values as previously described. As LVMVR is the earliest ventricular remodeling response to increased cardiac stress and afterload as well as its independence from the distorting effects of allometric assumptions in indexing, this was used as the index variable with which to examine the effects of atherosclerosis and arteriosclerosis on cardiac remodeling [16].

### Compliance calculation

Arteriosclerosis was measured using total arterial compliance (TAC). This was calculated using the brachial pulse pressure (systolic blood pressure – diastolic blood pressure) and the CMR calculated indexed stroke volume using the following equation:

$$ Compliance=\frac{Pulse pressure}{indexed Stroke Volume} $$

### Statistical analysis

Data are expressed as mean ± standard deviation (SD) for continuous variables, median (range) for ordinal variables and N (%) for nominal variables. Normality tests were performed; if the test failed, where possible standard transformations such as square root, reciprocal or logarithmic transforms were used to generate a Gaussian distribution. To test the null hypothesis that samples originate from the same population an independent t-test was used for continuous variables. Chi-square or Fisher exact test were used as appropriate to compare nominal data. Spearman rank correlation co-efficient was used to determine the association between TAC, SAS and LVMVR. Multivariable linear regression analyses were performed for TAC, SAS, and LVMVR. For all 3 linear regressions the dependant variable (TAC, SAS, and LVMVR in turn) were log transformed (a constant of 1 was added to the SAS before log transformation to avoid logging values of 0). Independence of residuals was confirmed in all instances using the Durbin-Watson statistic. Linearity was confirmed using scatter plots, as was homoscedasticity as assessed by visual inspection of a plot of studentised residuals versus unstandardized predicted values. In all 3 linear regression models there was significant collinearity of cholesterol and LDL, therefore total cholesterol was excluded from the multiple linear regression model. The Scottish Index of Multiple Deprivation (SIMD) [17] was treated as a continuous variable within the model. Due to the known significant differences in cardiac mass and volumes between genders, analysis was conducted separately for men and women [18]. As the hypertension guidelines have changed substantially since the study was conceived when a blood pressure of < 145/95 was considered normotensive, a sensitivity analysis was performed to determine if the results hold true in a cohort who would continue to be considered normotensive according to the 2017 AHA/ACC guidelines (BP < 120/80) [19]. All data were analysed using SPSS (version 21.0, International Business Machines, Inc., Armonk, New York, USA. Significance was assumed when *p* < 0.05.

## Results

Of the 2047 participants eligible for and offered a CMR scan, 1528 (74.8% of those invited) completed or partially completed the scan protocol with 13 (1%) excluded due to missing or incomplete data, leaving 1515 in the final analysis. 373 (18.2%) did not consent for a scan and 12 (0.6%) failed to attend their CMR scan appointment. 101 participants (4.9%) were not scanned due to claustrophobia (*n* = 83), large body habitus (*n* = 3), inability to establish venous access/other technical issues (*n* = 15), or being unsafe to scan due to presence of metalwork (*n* = 34).

Of the final 1515, 574 were male (53.8 ± 8.2 years old) and 941 were female (54.3 ± 8.4 years old). The male population had a significantly higher systolic and diastolic blood pressure, a higher body mass index (BMI), a lower total cholesterol driven by lower HDL-cholesterol, higher random blood glucose, lower BNP, higher LV mass, LVMVR and compliance (p < 0.05 for all) (Table 1). There was no significant difference in SAS between males and females.

**Table 1:** Characteristics of the total cohort and by sex

	Total	Men	Women	*p*-value
N	1515	574	941	
Age (years)	54.1 ± 8.3	53.8 ± 8.2	54.3 ± 8.4	0.24
Heart rate (bpm)	63.4 ± 10.0	61.4 ± 9.7	64.6 ± 9.9	**< 0.001**
Systolic BP (mmHg)	122 ± 12	125 ± 11	121 ± 12.5	**< 0.001**
Diastolic BP (mmHg)	73 ± 9	75 ± 9	72 ± 9	**< 0.001**
BMI (kg/m^2^)	26.8 ± 4.3	27.1 ± 3.6	26.6 ± 4.6	**0.025**
Current smoker (%)	165 (10.9%)	51 (8.9%)	114 (12.1%)	0.057
Ex smoker (%)	414 (27.4%)	162 (28.3%)	252 (26.8%)	0.52
Non-smoker (%)	935 (61.8%)	360 (62.8%)	575 (61.1%)	0.53
Smoking pack years	6.03 ± 11.9	6.64 ± 13.7	5.65 ± 10.6	0.12
Family history of CVD	387 (25.5%)	131 (22.8%)	256 (27.2%)	0.058
Total cholesterol (mmol/L)	5.48 ± 0.97	5.40 ± 0.92	5.53 ± 1.0	**0.016**
LDL-Cholesterol (mmol/L)	3.40 ± 0.88	3.40 ± 0.83	3.39 ± 0.90	0.83
HDL-Cholesterol (mmol/L)	1.44 ± 0.43	1.25 ± 0.39	1.56 ± 0.41	**< 0.001**
Triglycerides (mmol/L)	1.47 ± 0.86	1.71 ± 0.96	1.32 ± 0.75	**< 0.001**
Random glucose (mmol/L)	5.18 ± 0.90	5.27 ± 0.78	5.11 ± 0.97	**0.018**
BNP (pg/ml)	27.4 ± 17.9	19.8 ± 13.7	32.1 ± 18.5	**< 0.001**
ASSIGN risk score (%)	9.3 ± 6.6	11.4 ± 6.5	7.9 ± 6.3	**< 0.001**
LVEDV (g/m^2^)	71.6 ± 12.4	77.3 ± 13.2	68.1 ± 10.5	**< 0.001**
LVESV (g/m^2^)	22.6 ± 7.1	25.0 ± 7.4	21.1 ± 6.6	**< 0.001**
LVSV (g/m^2^)	49.0 ± 8.3	52.2 ± 8.9	47.0 ± 7.2	**< 0.001**
LVEF (%)	68.8 ± 6.5	67.9 ± 6.2	69.3 ± 6.6	**< 0.001**
LV mass (g/m^2^)	55.1 ± 11.6	64.3 ± 10.6	49.5 ± 8.0	**< 0.001**
LVMVR (g/ml)	0.78 ± 0.15	0.85 ± 0.16	0.74 ± 0.13	**< 0.001**
Compliance (ml/m^2^/mmHg)	1.03 ± 0.28	1.17 ± 1.96	0.99 ± 0.27	**< 0.001**
SAS	1.27 ± 2.1	1.17 ± 1.96	1.34 ± 2.18	0.14
SIMD				
1	65 (4.3%)	20 (2.1%)	45 (4.8%)	0.72
2	78 (5.2%)	25 (4.4%)	53 (5.7%)	
3	101 (6.7%)	36 (6.3%)	65 (6.9%)	
4	77 (5.1%)	34 (5.9%)	43 (4.6%)	
5	93 (6.2%)	34 (5.9%)	59 (6.3%)	
6	165 (10.9%)	63 (11%)	102 (10.9%)	
7	246 (16.3%)	88 (15.3%)	158 (16.8%)	
8	295 (19.5%)	115 (20%)	180 (19.2%)	
9	275 (18.2%)	109 (19%)	166 (17.7%)	
10	117 (7.7%)	50 (3.3%)	67 (7.1%)	

*ASSIGN* Assessing cardiovascular risk using SIGN guidelines, *BMI* body mass index, *BNP* brain natriuretic peptide, *BP* blood pressure, *CVD* cardiovascular disease, *HDL* high density lipoprotein, *LDL* low density lipoprotein, *LVEDV* left ventricular end diastolic volume, *LVESV* left ventricular end systolic volume, *LVSV* left ventricular stroke volume, *LVEF* left ventricular ejection fraction, *LVMVR* left ventricular mass to volume ratio, *SAS* standardised atheroma score, *SIMD* Scottish Index of Multiple Deprivation

*P*-values < 0.05 highlighted in bold

### Arteriosclerosis

**Fig. 3:**
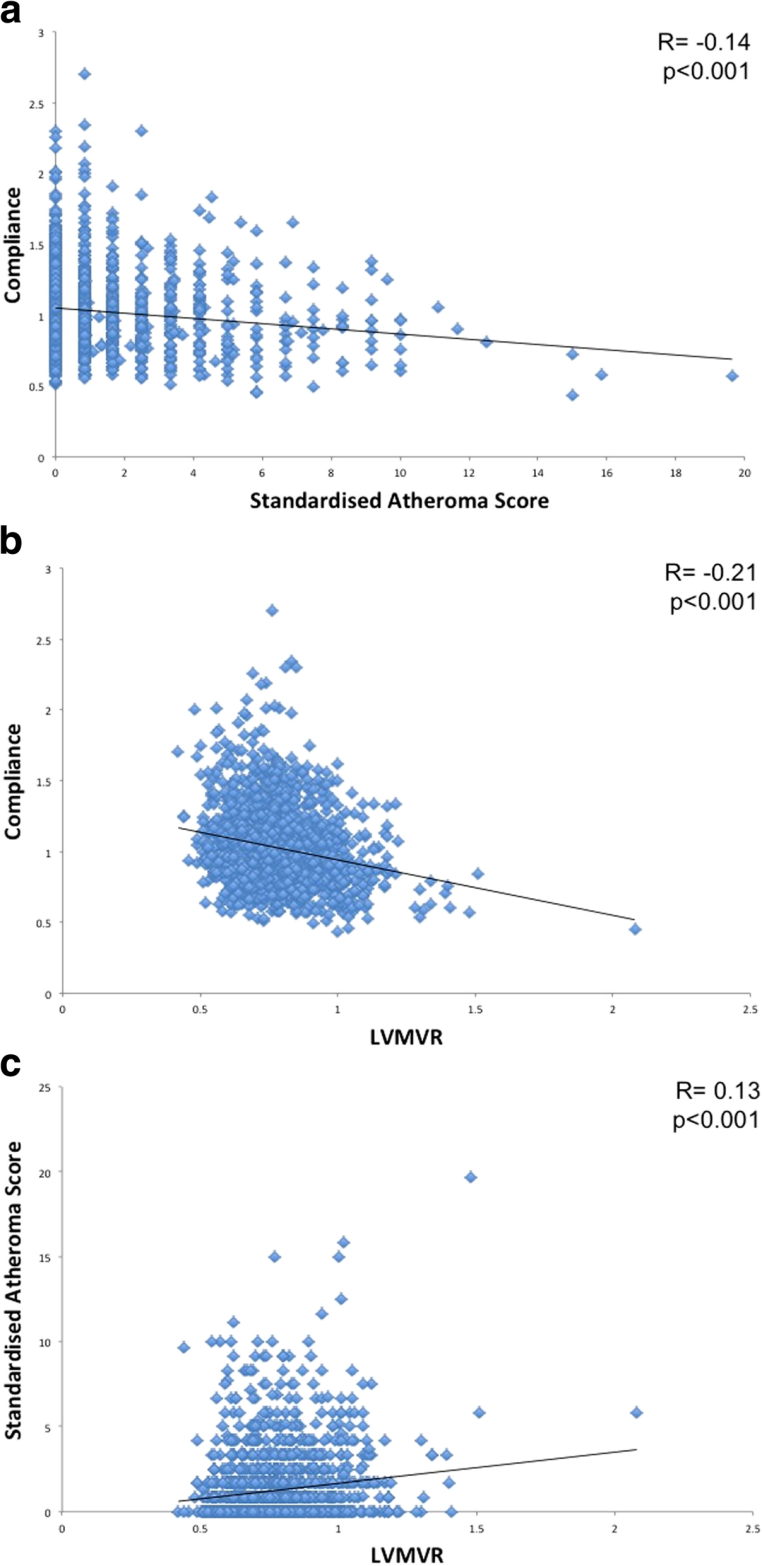
Scatter plots of: **a** Compliance against SAS; **b** Compliance against LVMVR; **c** SAS against LVMVR

In men: age, heart rate, systolic and diastolic blood pressure, low density lipoprotein (LDL) cholesterol, and socioeconomic deprivation were independently associated with TAC (model R^2^ = 0.67, *p* < 0.001). In women: age, heart rate, systolic and diastolic blood pressure, BMI, triglycerides, and smoking pack years were independently associated with TAC (model R^2^ = 0.67, *p* < 0.001) (See Table 2 for full results of multivariable linear regression). While there was a mild correlation between TAC and SAS (see Fig. 3), in neither males nor females was there a significant independent association between TAC and SAS. In the subgroup meeting current normotensive criteria only the associations between TAC and heart rate, systolic and diastolic blood pressure remained for both males and females (Additional file 1: Table S1).

**Table 2:** Backward multivariable linear regression of (log) TAC for each sex

	Men		Women	
	B (95% CI)	p	B (95% CI)	p
Age (years)	−0.001 (− 0.002- -0.000)	**0.004**	− 0.001 (− 0.002- -0.001)	**< 0.001**
Heart rate (bpm)	− 0.003 (− 0.003 - − 0.002)	**< 0.001**	-0.002 (− 0.002 - -0.001)	**< 0.001**
Systolic BP (mmHg)	−0.008 (− 0.009- − 0.008)	**< 0.001**	-0.008 (− 0.009- -0.008)	**< 0.001**
Diastolic BP (mmHg)	0.009 (0.008–0.010)	**< 0.001**	0.009 (0.008–0.009)	**< 0.001**
BMI (kg/m^2^)	0.001 (− 0.002–0.001)	0.62	−0.001 (− 0.002–0.000)	**0.028**
LDL-Cholesterol (mmol/L)	− 0.007 (− 0.014–0.000)	**0.047**	−0.005 (− 0.010–0.001)	0.078
HDL-Cholesterol (mmol/L)	0.016 (− 0.002–0.033)	0.077	0.000 (− 0.12–0.12)	0.95
Triglycerides (mmol/L)	−0.002 (− 0.009–0.006)	0.67	−0.009 (− 0.016 - -0.002)	**0.011**
Smoking status	0.001 (−0.010–0.013)	0.80	0.005 (− 0.004–0.013)	0.29
Pack years	0.000 (−0.001–0.00)	0.56	− 0.001 (− 0.001–0.00)	**0.011**
Family history of CVD	− 0.009 (− 0.023–0.005)	0.23	0.005 (− 0.005–0.015)	0.36
SIMD	−0.003 (− 0.005- − 0.001)	**0.016**	0.001 (− 0.001–0.003)	0.40
SAS	−0.001 (− 0.004–0.002)	0.62	0.000 (− 0.002–0.002)	0.78
Model R^2^	0.67	**< 0.001**	0.67	**< 0.001**

*BMI* body mass index, *BP* blood pressure, *CVD* cardiovascular disease, *HDL* high density lipoprotein, *LDL* low density lipoprotein, *SAS* standardised atheroma score, *SIMD* Scottish Index of Multiple Deprivation

*P*-values < 0.05 highlighted in bold

### Atherosclerosis

In men: age, BMI, and smoking pack-years were independently associated with the SAS (model R^2^ = 0.17, *p* < 0.001). In women: age and smoking pack years were independently associated with the SAS (model R^2^ = 0.67, *p* < 0.001) (See Table 3 for full results of multivariable linear regression). In neither males nor females was there a significant independent association between SAS and TAC. In the subgroup meeting current normotensive criteria only the associations between SAS and age and smoking pack years remained for both males and females (Additional file 1: Table S2).

**Table 3:** Backward multivariable linear regression of (Log) SAS

	Men		Women	
	B (95% CI)	p	B (95% CI)	p
Age (years)	0.01 (0.007–0.013)	**< 0.001**	0.008 (0.005–0.01)	**< 0.001**
Heart rate (bpm)	− 0.001 (− 0.003–0.002)	0.50	0.002 (− 0.001–0.004)	0.14
Systolic BP (mmHg)	0.002 (− 0.002–0.003)	0.41	0.002 (− 0.001–0.005)	0.17
Diastolic BP (mmHg)	0.000 (− 0.005–0.004)	0.87	-0.001 (− 0.005–0.002)	0.49
BMI (kg/m^2^)	− 0.006 (− 0.013–0.001)	0.11	−0.003 (− 0.007–0.002)	0.22
LDL-Cholesterol (mmol/L)	0.02 (− 0.006–0.005)	0.12	0.027 (0.004–0.05)	**0.022**
HDL-Cholesterol (mmol/L)	−0.03 (− 0.10–0.036)	0.35	0.010 (− 0.042–0.061)	0.72
Triglycerides (mmol/L)	0.02 (− 0.01–0.05)	0.19	0.007 (− 0.024–0.037)	0.67
Smoking status	0.038 (− 0.008–0.083)	0.10	−0.003 (− 0.041–0.035)	0.87
Pack years	0.003 (0.000–0.005)	**0.022**	0.004 (0.001–0.006)	**0.002**
Family history of CVD	0.04 (−0.016–0.097)	0.16	0.023 (−0.020–0.067)	0.29
SIMD	−0.009 (− 0.019–0.001)	0.069	−0.003 (− 0.011–0.004)	0.40
Compliance	0.005 (− 0.12–0.13)	0.93	0.02 (− 0.10–0.14)	0.75
Model R^2^	0.16	**< 0.001**	0.11	**< 0.001**

*BMI* body mass index, *BP* blood pressure, *CVD* cardiovascular disease, *HDL* high density lipoprotein, *LDL* low density lipoprotein, *SAS* standardised atheroma score, *SIMD* Scottish Index of Multiple Deprivation

*P*-values < 0.05 highlighted in bold

### Left ventricular remodelling

In men: systolic and diastolic blood pressure, current smoking status, BMI, TAC and SAS were independently associated with LVMVR (model R^2^ = 0.19, p < 0.001). In women: age, systolic and diastolic blood pressure, LDL-cholesterol, current smoking status, smoking pack years, BMI and TAC were independently associated with LVMVR (model R^2^ = 0.23, p < 0.001) (See Table 4 for full results of multivariable linear regression). In the subgroup meeting current normotensive criteria, the majority of these associations was lost in men with only TAC remaining as the only significant correlation with LVMVR, in women the association between LVMVR and age, systolic and diastolic blood pressure, LDL-cholesterol, BMI and TAC remained (Additional file 1: Table S3).

**Table 4:** Multivariable linear regression of (log) left ventricular mass to volume ratio

	Men		Women	
	B	p	B (95% CI)	p
Age (years)	−0.001 (− 0.001–0.00)	0.14	0.001 (0.00–0.002)	**0.012**
Heart rate (bpm)	0.00 (−0.001–0.001)	0.94	0.00 (− 0.001–0.00)	0.49
Systolic BP (mmHg)	−0.002 (− 0.003- − 0.001)	**< 0.001**	-0.001 (− 0.002- -0.001)	**< 0.001**
Diastolic BP (mmHg)	0.003 (0.002–0.004)	**< 0.001**	0.004 (0.003–0.004)	**< 0.001**
BMI (kg/m^2^)	0.005 (− 0.003–0.012)	0.20	0.008 (0.002–0.013)	**0.008**
LDL-Cholesterol (mmol/L)	0.01 (−0.008–0.028)	0.30	− 0.008 (− 0.02–0.005)	0.23
HDL-Cholesterol (mmol/L)	−0.001 (− 0.009–0.007)	0.79	0.003 (− 0.005–0.01)	0.46
Triglycerides (mmol/L)	0.015 (0.003–0.027)	**0.012**	0.018 (0.008–0.027)	**< 0.001**
Smoking status	0.00 (−0.001–0.001)	0.85	−0.001 (− 0.001–0.00)	**0.033**
Pack years	0.003 (0.001–0.005)	**0.001**	0.001 (0.00–0.002)	**0.028**
Family history of CVD	−0.003 (− 0.018–0.012)	0.70	0.002 (− 0.008–0.013)	0.69
SIMD	0.001 (− 0.001–0.004)	0.31	0.001 (− 0.001–0.003)	0.34
Compliance	− 0.12 (− 0.16- -0.091)	**< 0.001**	−0.12 (− 0.15- -0.086)	**< 0.001**
SAS	0.005 (0.002–0.008)	**0.002**	0.002 (0.00–0.004)	0.062
Model R^2^	0.19	**< 0.001**	0.23	**< 0.001**

*BMI* body mass index, *BP* blood pressure, *CVD* cardiovascular disease, *HDL* high density lipoprotein, *LDL* low density lipoprotein, *SAS* standardised atheroma score, *SIMD* Scottish Index of Multiple Deprivation

*P*-values < 0.05 highlighted in bold

## Discussion

In the current study we have shown: 1) that arteriosclerosis and atherosclerosis have different risk factors; 2) that when cardiovascular risk factors are accounted for, arteriosclerosis and atherosclerosis show no significant association with each other; and 3) arteriosclerosis is associated with early LV remodelling.

Studies examining the link between atherosclerosis and arteriosclerosis have produced mixed results. The Rotterdam study showed a significant link between pulse wave velocity (PWV) – a measure of arterial stiffening - and aortic arterial calcification [20], however a twin study showed that while PWV was associated with aortic wall calcification this was mediated by genetic factors and that PWV was not linked to plaque burden [21]. Previous work in 306 seventy-year-olds in the PIVUS (Prospective Investigation of the Vasculature in Uppsala Seniors) study found a correlation between the total atherosclerotic burden as measured on whole body angiography and the carotid arterial distensibility and the stroke-volume to pulse pressure ratio [22]. However this study suffered from several limitations: it did not stratify by sex, a known confounder for stroke volume and pulse pressure; and did not index the stroke volume thus opening the study to significant confounding from body size [18]. Indeed previous work has shown compliance calculated using indexed SV but not unadjusted SV to be an independent predictor for cardiovascular events demonstrating the importance of these adjustments [23]. A study by Sawabe et al. who conducted post mortem examinations in 304 individuals with a pre mortem PWV showed a weak correlation between the PWV and atherosclerotic plaque burden (spearman rho = 0.32) which persisted after accounting for age and systolic blood pressure, but did not attempt to account for other risk factors [24]. Thus the lack of correlation in our current study compared to those of the Sawabe group may be due to a lack of correction for other confounding variables such as BMI or smoking both of which we have shown to be correlated with arteriosclerosis and atherosclerosis respectively. Further supportive evidence of our results comes from a previous study on coronary plaque volume on intravascular ultrasound showing no significant correlation with carotid femoral pulse wave velocity [25]. Similarly another Rotterdam study showed a significant correlation between carotid plaque and aortic PWV which was lost after adjusting for cardiovascular risk factors [6]. Limitations of these latter studies is their correlation of one regional measure of arterial stiffness with plaque volume measured in a separate region, however atherosclerosis in one region poorly correlates with disease in other regions [8, 26]. In addition these studies examined populations at high risk for or with known cardiovascular disease. This is the first study to look at those without established cardiovascular disease and without high risk for the development of cardiovascular disease providing key insights into early disease formation. Thus it is feasible that in advanced disease, increased arterial stiffness could interact with existing plaque to accelerate its growth or reduce its stability.

The finding of a significant correlation between systemic arterial stiffness and left ventricular remodeling is in agreement with that of MESA (MultiEthnic Study of Atherosclerosis), who also found a significant increase in the LVMVR ratio with increasing arterial stiffness [27]. Given the known importance of the LVMVR in its prediction of future cardiovascular events [28], this provides further weight to this being a significant mechanism by which arterial stiffness exerts its effects on future cardiovascular risk.

Our systemic approach to the assessment of these two separate entities and our observations are both novel and useful to our understanding of cardiovascular disease, especially as we have focused on those with low-intermediate cardiovascular risk before significant disease has developed. Age, blood pressure, BMI, cholesterol and smoking are all extremely well established risk factors and are central to cardiovascular risk stratification for primary prevention strategies [29, 30]. It is therefore interesting that out of these risk factors, one (blood pressure) strongly predicts arterial stiffening, one (smoking) predicts plaque formation and another (BMI) predicts early cardiac remodeling. Given that all are strong risk factors for future cardiovascular events this study suggests that they might all exert this risk through independent, separate mechanisms although further longitudinal studies are required to determine causation. Planned long term follow-up in the TASCFORCE study will provide a key opportunity for examining these further.

Our study suffers from several limitations. Whole body angiography is a lumenographic technique, thus potentially misses the earliest stages of atherosclerosis of fatty streaks along the vascular lumen, as well as requiring the administration of intravenous contrast [31]. However no other technique currently exists for the simultaneous acquisition of the whole body vasculature. Techniques such as 3D–black-blood imaging, which can also be performed without contrast, hold potential for this but to date have only been utilized at single vessel sites [32]. In addition it is arguable that since clinical manifestations of atherosclerosis are secondary to plaque stenosis or rupture rather than simple fatty streaks, that this is the most clinically relevant association to examine. Participants were recruited based on a previous higher threshold for the diagnosis of hypertension (145/90 mmHg) and using the ATP-III criteria for cardiovascular disease 10 year risk, both of which were the reference standards at the point of study design, but have been superseded by more contemporaneous risk criteria and thresholds. Thus our ‘healthy cohort’ included some participants with what would now be considered hypertension, potentially confounding associations between stiffness, blood pressure and left ventricular remodeling, although these persisted on multiple regression models. The pulse pressure for compliance measurement was derived from the brachial blood pressure measurement rather than use of a central measurement or pulse wave analysis which is a more accurate for the estimation of this [33]. However, estimation of TAC using blood pressure derived from a brachial sphygmomanometer has consistently proven to be an independent predictor of future cardiovascular events in multiple cohorts [23, 34, 35], and the observed associations between compliance and cardiovascular risk factors are consistent with the literature using both a peripheral pressure measurement and PWV [36]. Finally the blood pressure was not acquired contemporaneously with the stroke volume. Diurnal variation and situational variations are known to affect blood pressure thus this could confound the calculation of compliance. The effect size of this is likely to be small however as if significant variation between within scan blood pressure and without scan blood pressure existed this would be most manifest in an obviation or significant reduction of the association between the compliance and blood pressure, yet these variables demonstrated the strongest correlation with one another on univariate and multivariate analysis.

## Conclusions

In conclusion systemic arteriosclerosis is associated with LVremodelling but not atherosclerosis. Future efforts in cardiovascular risk prevention should thus seek to address both arteriosclerosis and atherosclerosis individually.

## Additional file

Additional file 1: Table S1. Backward multivariable linear regression of (log) TAC for males and females in those with normal blood pressure (systolic blood pressure < 120 mmHg and diastolic blood pressure < 80 mmHg). **Table S2.** Backward multivariable linear regression of (Log) SAS in those with normal blood pressure (systolic blood pressure < 120 mmHg and diastolic blood pressure < 80 mmHg). **Table S3.** Multivariable linear regression of (log) LVMVR in those with normal blood pressure (systolic blood pressure < 120 mmHg and diastolic blood pressure < 80 mmHg). **Figure S1.** Scatter plots of BMI against LVMVR. (DOCX 152 kb)

## Abbreviations

BMI: Body mass index
BNP: Brain natriuretic peptide
CMR: Cardiovascular magnetic resonance
LV: Left ventricle/Left ventricular
LVMVR: Left ventricular mass to volume ratio
PWV: Pulse wave velocity
SAS: Standardised atheroma score
SIMD: Scottish Index of Multiple Deprivation
TAC: Total arterial compliance
WB-CMRA: Whole body cardiovascular magnetic resonance angiography

## References

[1] Ben-Shlomo Y, Spears M, Boustred C, May M, Anderson SG, Benjamin EJ, Boutouyrie P, Cameron J, Chen CH, Cruickshank JK, Hwang SJ, Lakatta EG, Laurent S, Maldonado J, Mitchell GF, Najjar SS, Newman AB, Ohishi M, Pannier B, Pereira T, Vasan RS, Shokawa T, Sutton-Tyrell K, Verbeke F, Wang KL, Webb DJ, Willum Hansen T, Zoungas S, CM ME, Cockcroft JR (2014). Aortic pulse wave velocity improves cardiovascular event prediction: an individual participant meta-analysis of prospective observational data from 17,635 subjects. J Am Coll Cardiol.

[2] Wilkinson IB, McEniery CM, Cockcroft JR (2009). Arteriosclerosis and atherosclerosis: guilty by association. Hypertension.

[3] Kolipaka A, Illapani VSP, Kalra P, Garcia J, Mo X, Markl M, White RD (2017). Quantification and comparison of 4D-flow MRI-derived wall shear stress and MRE-derived wall stiffness of the abdominal aorta. J Magn Reson Imaging.

[4] Megnien JL, Simon A, Denarie N, Del-Pino M, Gariepy J, Segond P, Levenson J (1998). Aortic stiffening does not predict coronary and extracoronary atherosclerosis in asymptomatic men at risk for cardiovascular disease. Am J Hypertens.

[5] Torii S, Arima H, Ohkubo T, Fujiyoshi A, Kadota A, Takashima N, Kadowaki S, Hisamatsu T, Saito Y, Miyagawa N, Zaid M, Murakami Y, Abbott RD, Horie M, Miura K, Ueshima H (2015). Association between pulse wave velocity and coronary artery calcification in Japanese men the Shiga epidemiological study of subclinical atherosclerosis (SESSA). J Atheroscler Thromb.

[6] Selwaness M, Van Den Bouwhuijsen Q, FUS M-R, Verwoert GC, Hofman A, Franco OH, JCM W, van der Lugt A, Vernooij MW, Wentzel JJ (2014). Arterial stiffness is associated with carotid intraplaque hemorrhage in the general population: the rotterdam study. Arterioscler Thromb Vasc Biol.

[7] Hansen T, Wikström J, Eriksson M-O, Lundberg A, Johansson L, Ljungman C, Hoogeven R, Ahlström H (2006). Whole-body magnetic resonance angiography of patients using a standard clinical scanner. Eur Radiol.

[8] Weir-McCall JR, Khan F, Lambert MA, Adamson CL, Gardner M, Gandy SJ, Ramkumar PG, Belch JJF, Struthers AD, Rauchhaus P, Morris AD, Houston JG (2014). Common carotid intima media thickness and ankle-brachial pressure index correlate with local but not global atheroma burden: a cross sectional study using whole body magnetic resonance angiography. PLoS One.

[9] Lundberg C, Johansson L, Barbier CE, Lind L, Ahlström H, Hansen T (2013). Total atherosclerotic burden by whole body magnetic resonance angiography predicts major adverse cardiovascular events. Atherosclerosis.

[10] Bamberg F, Parhofer KG, Lochner E, Marcus RP, Theisen D, Findeisen HM, Hoffmann U, Schönberg SO, Schlett CL, Reiser MF, Weckbach S (2013). Diabetes mellitus: long-term prognostic value of whole-body MR imaging for the occurrence of cardiac and cerebrovascular events. Radiology.

[11] Bertheau RC, Bamberg F, Lochner E, Findeisen HM, Parhofer KG, Kauczor H-U, Schoenberg SO, Weckbach S, Schlett CL (2016). Whole-body MR imaging including angiography: predicting recurrent events in diabetics. Eur Radiol.

[12] Weir-McCall JR, Yeap PM, Papagiorcopulo C, Fitzgerald K, Gandy SJ, Lambert M, Belch JJF, Cavin I, Littleford R, Macfarlane JA, Matthew SZ, Nicholas RS, Struthers AD, Sullivan F, Waugh SA, White RD, Houston JG (2016). Left ventricular noncompaction. Anatomical Phenotype or Distinct Cardiomyopathy? J Am Coll Cardiol.

[13] Expert Panel on Detection, Evaluation and T of HBC in A (2001). Executive summary of the third report of the National Cholesterol Education Program (NCEP) expert panel on detection, evaluation, and treatment of high blood cholesterol in adults (adult treatment panel III). JAMA.

[14] Gandy SJ, Lambert M, Belch JJF, Cavin ID, Crowe E, Littleford R, Macfarlane JA, Matthew SZ, Martin P, Nicholas RS, Struthers AD, Sullivan F, Waugh SA, White RD, Weir-McCall JR, Houston JG (2015). Technical assessment of whole body angiography and cardiac function within a single MRI examination. Clin Radiol.

[15] Duce SL, Weir-McCall JR, Gandy SJ, Matthew SZ, Cassidy DB, McCormick L, Rauchhaus P, Looker H, Colhoun HM, Houston JG (2015). Cohort comparison study of cardiac disease and atherosclerotic burden in type 2 diabetic adults using whole body cardiovascular magnetic resonance imaging. Cardiovasc Diabetol.

[16] Rodrigues JCL, Amadu AM, Dastidar AG, Szantho GV, Lyen SM, Godsave C, Ratcliffe LEK, Burchell AE, Hart EC, Hamilton MCK, Nightingale AK, Paton JFR, Manghat NE, Bucciarelli-Ducci C (2016). Comprehensive characterisation of hypertensive heart disease left ventricular phenotypes. Heart.

[17] Woodward M, Brindle P, Tunstall-Pedoe H (2007). SIGN group on risk estimation: adding social deprivation and family history to cardiovascular risk assessment: the ASSIGN score from the Scottish heart health extended cohort (SHHEC). Heart.

[18] Gandy SJ, Lambert M, Belch J, Cavin I, Crowe E, Littleford R, MacFarlane JA, Matthew SZ, Martin P, Nicholas RS, Struthers A, Sullivan F, Waugh SA, White RD, Weir-McCall JR, Houston JG (2016). 3T MRI investigation of cardiac left ventricular structure and function in a UK population: the tayside screening for the prevention of cardiac events (TASCFORCE) study. J Magn Reson Imaging.

[19] Whelton PK, Committee W, Carey RM, Chair V, Aronow WS, Committee Member W, Casey DE, Collins KJ, Dennison Himmelfarb C, DePalma SM, Gidding S, Jamerson KA, Jones DW, MacLaughlin EJ, Muntner P, Ovbiagele B, Smith SC, Spencer CC, Stafford RS, Taler SJ, Thomas RJ, Williams KA, Williamson JD, Wright JT: 2017 ACC/AHA/AAPA/ABC/ACPM/AGS/APhA/ASH/ASPC/NMA/PCNA guideline for the prevention, detection, evaluation, and Management of High Blood Pressure in adults. J Am Coll Cardiol 2017:735–1097.

[20] van Popele NM, Grobbee DE, Bots ML, Asmar R, Topouchian J, Reneman RS, PG HA, van der Kuip DAM, Hofman A, JCM W (2001). Association between arterial stiffness and atherosclerosis: the Rotterdam study. Stroke.

[21] Cecelja M, Hussain T, Greil G, Botnar R, Preston R, Moayyeri A, Spector TD, Chowienczyk P (2013). Multimodality imaging of subclinical aortic atherosclerosis: relation of aortic stiffness to calcification and plaque in female twins. Hypertension.

[22] Lind L, Andersson J, Hansen T, Johansson L, Ahlström H. Atherosclerosis measured by whole body magnetic resonance angiography and carotid artery ultrasound is related to arterial compliance, but not to endothelium-dependent vasodilation - the prospective investigation of the vasculature in Uppsala seniors (PIV. Clin Physiol Funct Imaging. 2009;29:321–9. 10.1111/j.1475-097X.2009.00871.x 19486081

[23] de Simone G, Roman MJ, Koren MJ, Mensah GA, Ganau A, Devereux RB (1999). Stroke volume/pulse pressure ratio and cardiovascular risk in arterial hypertension. Hypertension.

[24] Sawabe M, Takahashi R, Matsushita S, Ozawa T, Arai T, Hamamatsu A, Nakahara KI, Chida K, Yamanouchi H, Murayama S, Tanaka N (2005). Aortic pulse wave velocity and the degree of atherosclerosis in the elderly: a pathological study based on 304 autopsy cases. Atherosclerosis.

[25] McLeod AL, Uren NG, Wilkinson IB, Webb DJ, Maxwell SRJ, Northridge DB, Newby DE (2004). Non-invasive measures of pulse wave velocity correlate with coronary arterial plaque load in humans. J Hypertens.

[26] van den Bosch HCM, Westenberg JJM, Setz-Pels W, Wondergem J, Wolterbeek R, Duijm LEM, Teijink JAW, de Roos A (2015). Site-specific association between distal aortic pulse wave velocity and peripheral arterial stenosis severity: a prospective cardiovascular magnetic resonance study. J Cardiovasc Magn Reson.

[27] Zamani P, Bluemke DA, Jacobs DR, Duprez DA, Kronmal R, Lilly SM, Ferrari VA, Townsend RR, Lima JA, Budoff M, Segers P, Hannan P, Chirinos JA (2015). Resistive and pulsatile arterial load as predictors of left ventricular mass and geometry the multi-ethnic study of atherosclerosis. Hypertension.

[28] Bluemke DA, Kronmal RA, JAC L, Liu K, Olson J, Burke GL, Folsom AR (2008). The relationship of left ventricular mass and geometry to incident cardiovascular events: the MESA (multi-ethnic study of atherosclerosis) study. J Am Coll Cardiol.

[29] Mancia G, Fagard R, Narkiewicz K, Redon J, Zanchetti A, Böhm M, Christiaens T, Cifkova R, De Backer G, Dominiczak A, Galderisi M, Grobbee DE, Jaarsma T, Kirchhof P, Kjeldsen SE, Laurent S, Manolis AJ, Nilsson PM, Ruilope LM, Schmieder RE, Sirnes PA, Sleight P, Viigimaa M, Waeber B, Zannad F, Burnier M, Ambrosioni E, Caufield M, Coca A, Olsen MH (2013). 2013 ESH/ESC guidelines for the management of arterial hypertension: the task force for the management of arterial hypertension of the European Society of Hypertension (ESH) and of the European Society of Cardiology (ESC). Eur Heart J.

[30] Bibbins-Domingo K, Grossman DC, Curry SJ, Davidson KW, Epling JW, García FAR, Gillman MW, Kemper AR, Krist AH, Kurth AE, Landefeld CS, LeFevre ML, Mangione CM, Phillips WR, Owens DK, Phipps MG, Pignone MP (2016). Statin use for the primary prevention of cardiovascular disease in adults. JAMA.

[31] Mitchell JR, Schwartz CJ (1962). Relationship between arterial disease in different sites. A study of the aorta and coronary, carotid, and iliac arteries Br Med J.

[32] Treitl KM, Maurus S, Sommer NN, Kooijman-Kurfuerst H, Coppenrath E, Treitl M, Czihal M, Hoffmann U, Dechant C, Schulze-Koops H, Saam T (2017). 3D-black-blood 3T-MRI for the diagnosis of thoracic large vessel vasculitis: a feasibility study. Eur Radiol.

[33] Cheng H-M, Sung S-H, Shih Y-T, Chuang S-Y, Yu W-C, Chen C-H (2012). Measurement of central aortic pulse pressure: noninvasive brachial cuff-based estimation by a transfer function vs. a novel pulse wave analysis method. Am J Hypertens.

[34] Maroules CD, Khera A, Ayers C, Goel A, Peshock RM, Abbara S, King KS (2014). Cardiovascular outcome associations among cardiovascular magnetic resonance measures of arterial stiffness: the Dallas heart study. J Cardiovasc Magn Reson.

[35] Mancusi C, Gerdts E, de Simone G, Midtbø H, Lønnebakken MT, Boman K, Wachtell K, Dahlöf B, Devereux RB (2017). Higher pulse pressure/stroke volume index is associated with impaired outcome in hypertensive patients with left ventricular hypertrophy the LIFE study. Blood Press.

[36] Lilly SM, Jacobs D, Bluemke DA, Duprez D, Zamani P, Chirinos J (2014). Resistive and pulsatile arterial hemodynamics and cardiovascular events: the multiethnic study of atherosclerosis. J Am Heart Assoc.

