# Supplementary material for: Systemic arteriosclerosis is associated with left ventricular remodeling but not atherosclerosis: a TASCFORCE study
Source: J Cardiovasc Magn Reson. 2018 Jan 30;20:7. doi: 10.1186/s12968-018-0428-0 (PMC5791244; doi:10.1186/s12968-018-0428-0)

**Systemic arteriosclerosis is associated with left ventricular remodeling but not atherosclerosis: a TASCFORCE study.**

Jonathan R Weir-McCall FRCR^1^, Matthew Lambert MBChB^1^, Stephen J Gandy PhD^2^, Jill JF Belch FRCP^1^, Ian Cavin PhD^2^, Shelley A Henderson PhD^2^, Roberta Littleford PhD^1^, Jennifer A Macfarlane PhD^2^, Shona Z Matthew PhD^1^, R Stephen Nicholas PhD^2^, Allan D Struthers FRCP^1^, Frank Sullivan FRSE^3^, Richard D White FRCR^4^, J Graeme Houston MD FRCR^1^.

**Table S1:** Backward multivariable linear regression of (log) TAC for males and females in those with normal blood pressure (systolic blood pressure <120mmHg and diastolic blood pressure <80mmHg).

|  | **Men** | | **Women** | |
| --- | --- | --- | --- | --- |
|  | B (95% CI) | **p** | B (95% CI) | **p** |
| N | 143 |  | 361 |  |
| Age (years) | 0.000 (-0.002- 0.002) | 0.98 | -0.001 (-0.002 - 0.000) | 0.17 |
| Heart rate (bpm) | -0.004 (-0.006 - -0.003) | **<0.001** | -0.002 (-0.003 - -0.001) | **<0.001** |
| Systolic BP (mmHg) | -0.009 (-0.011- -0.007) | **<0.001** | -0.009 (-0.010- -0.008) | **<0.001** |
| Diastolic BP (mmHg) | 0.010 (0.008-0.012) | **<0.001** | 0.011 (0.010-0.012) | **<0.001** |
| BMI (kg/m^2^) | -0.002 (-0.006-0.002) | 0.33 | -0.002 (-0.003 - 0.000) | 0.058 |
| LDL-Cholesterol (mmol/L) | -0.003 (-0.018 - 0.012) | 0.67 | -0.007 (-0.016 - 0.001) | 0.09 |
| HDL-Cholesterol (mmol/L) | -0.008 (-0.044- 0.028) | 0.68 | -0.014 (-0.034- 0.005) | 0.14 |
| Triglycerides (mmol/L) | 0.000 (-0.017 - -0.018) | 0.96 | -0.010 (-0.021 - 0.000) | 0.058 |
| Smoking status | 0.003 (-0.020-0.026) | 0.78 | -0.001 (-0.014 - 0.012) | 0.89 |
| Pack years | 0.000 (-0.001-0.002) | 0.86 | 0.000 (-0.001 - 0.001) | 0.52 |
| FHx of CVD | -0.010 (-0.040-0.019) | 0.50 | 0.009 (-0.006 - 0.024) | 0.25 |
| SIMD | -0.003 (-0.008- -0.002) | 0.25 | 0.002 (-0.001-0.005) | 0.20 |
| SAS | -0.002 (-0.011-0.006) | 0.62 | 0.003 (-0.001-0.007) | 0.15 |
| Model R^2^ | 0.63 | **<0.001** | 0.63 | **<0.001** |

**Table S2:** Backward multivariable linear regression of (Log) SAS in those with normal blood pressure (systolic blood pressure <120mmHg and diastolic blood pressure <80mmHg).

|  | **Men** | | **Women** | |
| --- | --- | --- | --- | --- |
| **n** | 143 |  | 361 |  |
|  | B (95% CI) | **p** | B (95% CI) | **p** |
| Age (years) | 0.009 (0.004-0.014) | **0.001** | 0.008 (0.004-0.013) | **<0.001** |
| Heart rate (bpm) | -0.003 (-0.008-0.002) | 0.22 | 0.001 (-0.002-0.005) | 0.51 |
| Systolic BP (mmHg) | 0.002 (-0.007-0.011) | 0.70 | 0.005 (-0.001-0.011) | 0.08 |
| Diastolic BP (mmHg) | 0.003 (-0.006-0.012) | 0.54 | -0.003 (-0.009-0.004) | 0.40 |
| BMI (kg/m^2^) | -0.005 (-0.019 -0.009) | 0.51 | -0.001 (-0.008-0.007) | 0.82 |
| LDL-Cholesterol (mmol/L) | 0.041 (-0.010-0.092) | 0.11 | 0.019 (-0.015-0.054) | 0.27 |
| HDL-Cholesterol (mmol/L) | 0.045 (-0.08 - 0.17) | 0.48 | -0.030 (-0.11-0.049) | 0.46 |
| Triglycerides (mmol/L) | -0.015 (-0.076 – 0.046) | 0.63 | 0.002 (-0.043-0.047) | 0.94 |
| Smoking status | 0.000 (-0.080-0.079) | 0.99 | -0.012 (-0.067-0.042) | 0.66 |
| Pack years | 0.006 (0.001-0.011) | **0.015** | 0.004 (0.000-0.008) | **0.04** |
| FHx of CVD | 0.06 (-0.042-0.16) | 0.25 | 0.016 (-0.047-0.078) | 0.62 |
| SIMD | 0.002 (-0.016-0.02) | 0.82 | -0.004 (-0.016-0.007) | 0.45 |
| Compliance | -0.063 (-0.36 - 0.24) | 0.68 | 0.17 (-0.055-0.39) | 0.14 |
| Model R^2^ | 0.22 | **0.001** | 0.10 | **0.001** |

**Table S3:** Multivariable linear regression of (log) LVMVR in those with normal blood pressure (systolic blood pressure <120mmHg and diastolic blood pressure <80mmHg).

|  | **Men** | | **Women** | |
| --- | --- | --- | --- | --- |
| **N** | **140** | | **354** | |
|  | B | **p** | B (95% CI) | **p** |
| Age (years) | -0.001 (-0.003-0.001) | 0.18 | 0.001 (0.00-0.002) | 0.24 |
| Heart rate (bpm) | 0.001 (-0.001-0.002) | 0.43 | 0.00 (-0.001-0.001) | 0.90 |
| Systolic BP (mmHg) | -0.002 (-0.005- -0.001) | 0.14 | -0.002 (-0.003- 0.000) | **0.017** |
| Diastolic BP (mmHg) | 0.001 (-0.002-0.003) | 0.56 | 0.003 (0.002-0.005) | **<0.001** |
| BMI (kg/m^2^) | 0.001 (-0.003-0.006) | 0.56 | 0.005 (0.003-0.007) | **<0.001** |
| LDL-Cholesterol (mmol/L) | 0.007 (-0.008- 0.023) | 0.37 | 0.017 (0.008- 0.025) | **<0.001** |
| HDL-Cholesterol (mmol/L) | -0.002 (-0.039-0.036) | 0.94 | -0.014 (-0.033-0.006) | 0.17 |
| Triglycerides (mmol/L) | 0.005 (-0.013-0.024) | 0.59 | -0.003 (-0.014-0.008) | 0.63 |
| Smoking status | 0.022 (-0.002-0.046) | 0.08 | 0.013 (0.00-0.027) | 0.054 |
| Pack years | -0.001 (-0.002-0.001) | 0.47 | -0.001 (-0.002-0.00) | 0.076 |
| FHx of CVD | -0.012 (-0.044-0.021) | 0.48 | -0.001 (-0.016-0.015) | 0.94 |
| SIMD | 0.000 (-0.005-0.005) | 0.98 | 0.00 (-0.003-0.002) | 0.73 |
| Compliance | -0.09 (-0.15- -0.026) | **0.006** | -0.12 (-0.18- -0.070) | **<0.001** |
| SAS | 0.008 (-0.001-0.018) | 0.082 | 0.001 (-0.003-0.005) | 0.53 |
| Model R^2^ | 0.19 | **0.016** | 0.23 | **<0.001** |

**Figure S1:** Scatter plots of BMI against LVMVR


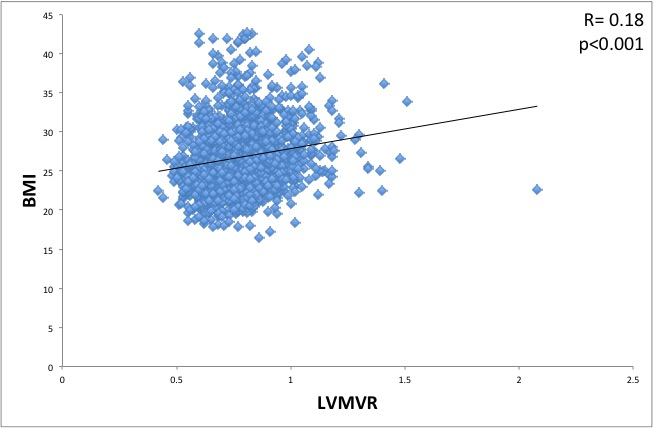

Supplement: Additional file 1: Table S1. — Backward multivariable linear regression of (log) TAC for males and females in those with normal blood pressure (systolic blood pressure < 120 mmHg and diastolic blood pressure < 80 mmHg). Table S2. Backward multivariable linear regression of (Log) SAS in those with normal blood pressure (systolic blood pressure < 120 mmHg and diastolic blood pressure < 80 mmHg). Table S3. Multivariable linear regression of (log) LVMVR in those with normal blood pressure (systolic blood pressure < 120 mmHg and diastolic blood pressure < 80 mmHg). Figure S1. Scatter plots of BMI against LVMVR. (DOCX 152 kb) [file 12968_2018_428_MOESM1_ESM.docx]
